# Recent updates in pediatric diffuse glioma classification: insights and conclusions from the WHO 5^th^ edition

**DOI:** 10.25122/jml-2023-0515

**Published:** 2024-07

**Authors:** Tarang Patel, Gyanendra Singh, Parth Goswami, Rushang Dave

**Affiliations:** 1 Department of Pathology, All India Institute of Medical Sciences, Rajkot, Gujarat, India; 2 Department of Pathology, Shantabaa Medical College and Hospital, Amreli, Gujarat, India

**Keywords:** WHO CNS 2021, diffuse astrocytoma, MYB/MYBL1 fusion, MAPK pathway alterations, angiocentric glioma, H3K27-altered, H3G34-mutant, hemispheric glioma, pediatric-type high-grade glioma

## Abstract

The World Health Organization (WHO) Central Nervous System (CNS) Tumors Classification 5^th^ edition (2021) integrates both molecular and histopathological criteria for diagnosing glial tumors. This updated classification highlights significant differences between pediatric and adult gliomas in terms of molecular characteristics and prognostic implications. The 5^th^ edition comprises a new category of pediatric-type diffuse low-grade glioma (PDLGG) and pediatric-type diffuse high-grade glioma (PDHGG), classified mainly based on genetic alterations and histopathological features. We reviewed the microscopy, diagnostic molecular pathology, and prognosis of various tumors under the categories PDLGG and PDHGG. The review also addresses the need for clarification concerning overlapping diagnostic features. PDLGG are characterized by diffuse growth, low-grade morphology, and MYB/MYBL1(MYB Proto-Oncogene Like 1) gene fusion or mitogen-activated protein kinase (MAPK) pathway alterations. In contrast, PDHGG is described by diffuse growth, high-grade morphology, and increased mitosis and often shows alterations of histone gene resulting in epigenetic alterations, which contrasts with common isocitrate dehydrogenase (IDH) mutation and epidermal growth factor receptor (EGFR) amplification seen in adult-type high-grade glioma.

## INTRODUCTION

Central nervous system (CNS) tumors are the most common solid tumors among children, with an incidence of 5.4 to 5.6 per 100,000 children [1]. About 45.7% of CNS tumor cases constitute glioma [2]. The World Health Organization (WHO) CNS Tumor Classification underwent significant updates in the 2021 5^th^ edition, evolving from the previous 4^th^ edition. The previous edition classified diffuse astrocytoma into three categories: isocitrate dehydrogenase (IDH)-mutant, IDH-wildtype, and not otherwise specified (NOS). WHO CNS 2021 categorized diffuse astrocytoma into adult-type and pediatric-type, with pediatric-type further divided into low-grade and high-grade categories.

Pediatric-type diffuse low-grade glioma (PDLGG) includes four subtypes with peculiar microscopic features and genetic alterations [3-6]. Recognition of key molecular changes identified by advanced molecular methods emphasizes the significance of pediatric-type diffuse high-grade glioma (PDHGG). This has led to the classification of PDHGG as a distinct group of tumors in the updated WHO classification [7-9].

This comprehensive review of PDLGG and PDHGG tumors highlights the importance of a combined approach, including molecular genetics and histopathology, for recent pathological classification, which is a requisite for accurate diagnosis, grading assignment, and further management.

### Classification

The WHO CNS 2021 classification builds on the previous WHO 2016 classification (revised 4^th^ edition) of CNS tumors, which was the first to integrate molecular details in diagnosing CNS tumors, moving beyond the conventional classification system based on histomorphology alone. The original concept behind the CNS 2016 classification was inspired by the Haarlem Consensus Guidelines [10] aimed at encompassing molecular information in CNS tumor diagnosis. The WHO CNS 2021 has incorporated suggestions from the 'Consortium to Inform Molecular and Practical Approaches to CNS Tumor Taxonomy (cIMPACT)’ [9,11-14] to combine molecular and morphological findings in the final diagnosis and grading of CNS tumors [8,15].

PDLGG are grouped due to their generally good prognoses despite diffuse growth. This category includes the following tumors: diffuse astrocytoma, MYB/MYBL1 altered, angiocentric glioma, polymorphous low-grade neuroepithelial tumor of the young (PLNTY), and diffuse low-grade glioma, mitogen-activated protein kinase (MAPK) pathway altered. Angiocentric glioma was previously classified under the 'other glioma’ category in the previous edition, while the remaining three tumors are newly added entities in the WHO CNS 2021 classification [16,17].

PDHGG are classified into four types in the latest 5^th^ edition of the WHO classification: diffuse midline glioma (DMG) H3 K27-altered, diffuse hemispheric glioma H3 G34-mutant, diffuse pediatric-type high-grade glioma H3-wildtype and IDH-wildtype, and infant-type hemispheric glioma [18].

### Clinical and radiological features

PDLGG are uncommon tumors predominantly seen in children and young adults, frequently presented with epilepsy [19]. Diffuse astrocytoma, altered by MYB/MYBL1 (MYB proto-oncogene like 1), is a cerebral tumor predominantly located in the cortical or subcortical areas. It can be associated with epilepsy, which may sometimes be drug-resistant, particularly in cases detected in childhood. Imaging characteristics include hypointensity on T1-weighted images, hyperintensity on T2-FLAIR, and no restricted diffusion on MRI [3]. Angiocentric glioma is seen in young adults with chronic partial seizures, commonly affecting the temporal lobe and often hyperintense on T2 and fluid-attenuated inversion recovery (FLAIR) radio-imaging [4,20]. Polymorphous low-grade neuroepithelial tumor of the young (PLNTY) is usually seen in the second and third decades of life, with temporal lobe involvement in 80% of cases. Clinically, patients present with partial complex epilepsy, and on MRI, they show central calcification, which is hypointense on T1/T2, and a non-calcified area that is hyperintense on T2 [5]. Diffuse low-grade astrocytoma, MAPK pathway altered is a childhood cerebral tumor, and on MRI, T2-FLAIR shows heterogenous signal lesions [6].

PDHGG is present among a wide age range from 2 to 65 years, and a median age of about 11-14 years has been reported in various studies. *H3-K27* altered (tri-methylation of lysine 27 on histone H3 protein) diffuse midline glioma (DMG) shows typical midline appearance including brainstem, hypothalamus, thalamus, pineal gland, and cerebellum. Diffuse intrinsic pontine glioma (DIPG) constitutes about 10-15% of all pediatric brain neoplasms and about 75% of all pediatric brainstem neoplasms. Most pontine cases present with the classic triad of cranial nerve palsy, pyramidal tract involvement, and ataxia [21]. Imaging reveals a sharply demarcated or diffuse growth pattern, variable contrast enhancement, and heterogeneous signal intensities on T1 and T2-weighted sequences, with areas of necrosis and hemorrhage [22]. Diffuse hemispheric glioma (DHG), H3G34-mutant can be isolated or may show multilobar involvement, predominantly involving frontal and parietal lobes. Clinical features may vary depending on the anatomical location, including seizures and motor or sensory deficiency symptoms [23]. MRI may show diffuse growth, necrosis, leptomeningeal spread, and peculiar contrast enhancement [22]. Diffuse pediatric-type high-grade glioma H3-wildtype and IDH-wildtype tumors are mostly localized in cerebral hemispheres and show motor or sensory deficit symptoms [24]. Radiology features include contrast enhancement, hypointensity on T1, T2/FLAIR-hyperintensity, indistinct borders, and peritumoral edema [22]. Infant-type hemispheric glioma is found predominantly in early childhood, and most cases occur within the first year of life. Presenting symptoms are acute and nonspecific [25]. Imaging reveals a superficial cerebral hemisphere location with possible areas of necrosis and large cystic structures [22].

### Histopathology and immunohistochemistry (IHC)

#### Diffuse astrocytoma, MYB/MYBL1 altered

*Histopathology:* This rare neoplasm features low-grade glial cells with round to spindle nuclei and a diffuse growth pattern within a fibrillary matrix. The cellularity may be similar to normal parenchyma, complicating its identification as a neoplasm. Entrapped neurons indicate the neoplastic process and a focal indistinct angiocentric pattern may be present in MYB-altered cases. Mitotic activity is minimal, with the absence of necrosis and microvascular proliferation.

*IHC:* Positive for glial fibrillary acidic protein (*GFAP*) and microtubule-associated protein 2 (*MAP2*) in entrapped neurons. Negative for *OLIG2, CD34*, and *IDH1 R132H* [3].

#### Angiocentric glioma

*Histopathology:* Characterized by a typical perivascular arrangement of monomorphic spindled glial cells arranged in a rosette-like pattern around blood vessels in single or multiple layers. An area of the myxoid or microcystic pattern may be seen. High-grade histological features are usually absent.

*IHC:* Similar to diffuse astrocytoma, MYB/MYBL1 altered, along with a few cases showing focal epithelial membrane antigen (*EMA*) positivity in a dot-like pattern representing epithelioid elements [4].

#### PLNTY

*Histopathology:* PLNTY is characterized by a diffuse growth pattern with oligodendroglioma-like areas. It shows round nuclei with perinuclear halo and branching capillary-sized blood vessels. Cells exhibit mild nuclear pleomorphism and nuclear membrane irregularities in the form of nuclear grooving and intranuclear inclusion. Coarse and confluent calcifications are seen in the majority of cases. Astrocytic elements may appear in fibrillary or spindled form. Changes in high-grade histology, gemistocytic areas, or microcystic elements are usually absent.

*IHC:* PLNTY is positive for *CD34* and *OLIG2*, unlike the previous two tumors. *CD34* may show diffuse intense positivity in tumor cells and background neural elements. PLNTY is negative for *EMA, IDH1*, and synaptophysin. A subset of cases shows *BRAF* immunoreactivity [5].

#### MAPK pathway-altered diffuse low-grade glioma with fibroblast growth factor 1 (FGFR1) involvement

*Histopathology:* It shows oligodendroglioma-like morphology without necrosis or microvascular proliferation.

*IHC:* Positive for *OLIG2*, unlike PLNTY, these tumors show restricted expression of *CD34* immunoreactivity. *BRAF* mutant variant presents diffusely arranged glial cells with bland nuclei and subpial clustering of tumor cells. These tumors lack high-grade morphology and are immunoreactive for *OLIG2* and *BRAF V600E* and negative for *IDH* markers [6].

#### Diffuse midline glioma (DMG), H3 K27-altered

*Histopathology:* Exhibits a diffuse growth pattern with small monomorphic cells, occasionally showing polymorphous differentiation into astrocytic, oligodendrocytic, epithelioid, piloid, giant cell, or undifferentiated pattern. These tumors may display mitotic activity with areas of microvascular proliferation and necrosis. However, they are considered WHO grade 4 tumors, irrespective of the foci of necrosis and vascular proliferation. The *EGFR* subtype is notable for frequent mitotic activity.

*IHC:* Positive for *OLIG2, MAP2*, and *S100*, whereas the EGFR-mutant subtype is negative for *OLIG2* and positive for *GFAP*. Positive nuclear staining for the H3 K27M antibody and negative nuclear staining for H3 K27me3 are helpful for the detection of scattered tumor cells amidst infiltrating areas. Enhancer of Zeste Homologs Inhibitory Protein (EZHIP) marker can underline EZHIP overexpression [21].

#### Diffuse Hemispheric Glioma (DHG), H3 G34-mutant

*Histopathology:* These cellular tumors show brisk mitosis with palisading necrosis and microvascular proliferation, resembling glioblastoma. Alternatively, tumor cells may be small with hyperchromatic nuclei resembling embryonal tumors.

*IHC:* It shows loss of alpha-thalassemia/mental retardation, X-linked (*ATRX*) expression, diffuse positivity for *p53*, and immunonegativity for *OLIG2*. The Ki-67 marker shows high positivity. They are also considered as a WHO Grade 4 tumor regardless of the presence of microvascular proliferation and necrosis. Antibodies are now available on IHC to detect G34R/V mutation [23]. The glioblastoma-like pattern is associated with high *GFAP* expression. An embryonal-like pattern is associated with diffuse synaptophysin and focal *GFAP*. However, both patterns show *OLIG2* negativity [18].

#### Diffuse pediatric-type high-grade glioma, H3-wildtype and IDH-wildtype

*Histopathology:* Diffuse pediatric-type high-grade glioma H3-wildtype and *IDH*-wildtype can exhibit glioblastoma-like or embryonal-type primitive morphology. The MYCN subtype may show a biphasic pattern with both diffuse pattern and circumscribed nodules having circumscription with normal brain parenchyma. Radiation-induced RTK1 subtype may show exuberant myxoid stroma in neoplasm.

*IHC:* It shows focal positivity for *GFAP* and *OLIG2*. The RTK1 subtype may show loss of *MSH2/MSH6*, corresponding with the germline mutation [24].

#### Infant-type hemispheric glioma

*Histopathology:* Infant-type hemispheric glioma shows high-grade morphology with high cellularity, nuclear pleomorphism, high mitosis, necrosis, and endothelial proliferation. Original diagnoses are often glioblastoma or desmoplastic infantile ganglioglioma/astrocytoma (DIG/DIA). Occasional gemistocytic differentiation is seen. Tumors with anaplastic lymphoma kinase (ALK) fusion may show ependymal differentiation or biphasic appearance (mixture of low-grade and high-grade components) or ganglion cell component.

*IHC:* Positive for *GFAP* and *OLIG2*, with *ALK* positivity in ALK fusion tumors [25].

### Molecular pathology

Diffuse astrocytoma, MYB/MYBL1 altered, shows amplifications and variants of *MYB* or *MYBL1*, with fusion involving partner genes like *PCDHGA1, MMP16*, and *MAMAL2. MYB* is a proto-oncogene involved in the proliferation and differentiation of cells. Angiocentric glioma shows typical fusion of *MYB* with partner gene *QKI*. Both tumors are negative for *IDH* and *H3* mutation [3,4,26].

PLNTY is associated with activation of the MAPK pathway, typically involving the *BRAF V600E* mutation and fusions with *FGFR2/FGFR3*. PLNTY tumors do not harbor *IDH* mutation or 1p/19q codeletion.

MAPK pathway-altered diffuse low-grade glioma manifests genetic alterations involving *BRAF* and *FGFR1* with occasional involvement of *FGFR2, FGFR3, NTRK1, NTRK2*, and *NTRK3*. They are negative for *IDH* and *H3* mutation and *CDKN2A* deletion [5,6].

The molecular profile of PDHGG is different from that of its adult counterpart. Compared to adult HGG, PDHGG has frequent *PDGFR, TP53*, and *K27M & G34* mutations on the *H3* histone. The core signaling pathways involved in PDHGG are the RTK/RAS/PI3K pathway, p53 pathway, and RB pathway [27].

Diffuse midline glioma (DMG), *H3* K27-altered, shows variable mutations in *H3F3A* and *HIST1H3B/HIST1H3C*, encoding histone *H3.3* and *H3.1*, leading to a methionine substitution at lysine at 28^th^ N-terminal position of histone [28]. *H3F3A* gene *H3.3* encoded K27M mutations are three times more common than the *H3.1* mutation [29]. The three subgroups responsible for H3K27me3 loss include *H3K27M* mutation, *EZHIP* overexpression, and *EGFR* mutation/overexpression [30]. All three subgroups have equal tumorigenic capability [31]. The most common mutation in *EGFR* is missense mutation of exon 7, followed by frame-shift mutation of exon 20 [28]. *H3K27* mutation may be associated with *BRAF V600E* mutation or rarely with *IDH1* mutation [18]. The association of *TP53* and *H3F3A* mutations in PDHGG is similar to *TP53* and *IDH* mutations in adult HGG [28,32]. Other pathways implicated in PDHGG pathogenesis are RTK/RAS/PI3K, TP53, and RB pathways [33]. A small subset of pediatric HGG is associated with mismatch repair (MMR) deficiency or Li-Fraumeni syndrome [34].

Diffuse hemispheric glioma H3 G34-mutant is characterized by a mutation in the *H3F3A* gene at codon 35, with the *G34R* mutation being more common than the *G34V* mutation. The *H3G34* mutation leads to decreased methylation of *H3K36* [35]. The tumor also shows a uniform genetic profile, including a *TP53* mutation and loss of *ATRX* (Alpha Thalassemia/Mental Retardation Syndrome X-Linked) expression. *ATRX* is necessary for the addition of *H3.3* at telomere ends and the silencing of those sites. Loss of *ATRX* function causes instability of the telomere end. It also shows chromosomal alterations in the form of loss of 4q and 2q [35]. PDHGG with GBM-like morphology shows PDGFRA mutation in one-third of cases [31]. Homozygous deletion of *CDKN2A* is found in nearly 75% of cases [36].

Diffuse pediatric-type high-grade glioma, *H3*-wildtype, and IDH-wildtype lack *IDH* and *H3* mutations. These tumors are categorized into three subtypes based on their DNA methylation profiles: *RTK1*, associated with platelet-derived growth factor receptor alpha (*PDGFRA*) amplification; tumors with Lynch syndrome or MMR syndrome are also of *RTK1* subtype; *RTK2*, associated with *EGFR* amplification and telomerase reverse transcriptase (*TERT*) promoter mutation, and *MYCN* associated with Myelocytomatosis-N (*MYCN*) amplification [23,37]. Some cases lacking these profiles are classified as 'NEC’ (Not Elsewhere Classified) per the latest WHO classification [32].

Infant-type hemispheric glioma shows gene rearrangements of *NTRK1/2/3* or *ROS/ALK1/MET* alterations, leading to fusion of any RTK having intracellular tyrosine kinase domain. Chromosomal microdeletion or copy number gain leads to the fusion of *ALK1* to various fusion partners [38,39]. *NTRK* gene fusions may lead to spindle cell morphology and a high Ki-67 index [40].

### Differential diagnosis

*MYB*/MYBL1 altered diffuse glioma and angiocentric glioma show overlapping microscopic morphology and are characterized by *MYB* gene involvement. The fusion partner gene is different in both tumors. Angiocentric glioma shows *MYB: QKI* fusion gene. Both tumors must be differentiated from adult-type diffuse glioma based on *IDH* mutation status [3,4].

PLNTY might be challenging to differentiate from oligodendroglioma in morphology. However, the latter is negative for *CD34* immunoreactivity and positive for *IDH* mutation and 1p/19q codeletion. MAPK-activated diffuse low-grade glioma with *FGFR1* alteration may overlap morphology with dysembryoplastic neuroepithelial tumor (DENT), PLNTY, and oligodendroglioma. Differentiating features are conspicuous histology of DENT and widespread *CD34* expression in PLNTY. The differential diagnosis for MAPK-activated and *BRAF* mutant variants are pilocytic astrocytoma, pleomorphic xanthoastrocytoma, ganglioglioma, and *H3-altered* diffuse midline glioma. They can be differentiated by observing histopathological features, IHC, and molecular signature markers [5,6].

Differential diagnosis of DMG includes pilocytic astrocytoma and ganglioglioma carrying *H3K27* mutation. The distinction is based on a morphological basis. Contrary to low-grade pediatric tumors, Rosenthal fibers and eosinophilic granular bodies are absent in DMG [41]. Pediatric infratentorial *IDH*-mutant glioma needs to be included in differential diagnosis, and differentiation is possible after testing for *IDH1* and *IDH2* sequencing [42]. *EGFR-mutant* DMG should be distinguished from tectal glioma based on histomorphology, wild-type *EGFR*, and frequent *KRAS BRAF* mutations [21]. *DHG, H3 G34-mutant* should be differentiated from adult high-grade glioma and other CNS embryonal tumors based on histopathology, IHC showing diffuse p53 positivity along with negativity for *ATRX* & *OLIG2* and molecular profile analysis [23,43].

*H3*-wildtype and *IDH*-wildtype gliomas are distinguished from diffuse midline gliomas (DMGs) by the retention of *H3K27me3*. To exclude infant-type hemispheric gliomas and desmoplastic infantile ganglioglioma/astrocytoma (DIG/DIA), molecular studies should be performed to check for *NTRK, ROS1, ALK*, and *MET* gene fusions. Adult glioblastomas can be differentiated by their characteristic morphology, *BRAF* mutations, *CDKN2A/CDKN2B* deletions, and chromosomal abnormalities such as +7/-10. Medulloblastomas and other embryonal tumors are less common in older children and can be excluded by testing for *OLIG2* negativity. Distinguishing radiation-induced cerebellar PDHGG of the *RTK1* subtype from a relapsed case of medulloblastoma can be particularly challenging [24,44].

For infant-type hemispheric glioma, the differential diagnosis may include DIG/DIA, supratentorial ependymoma, astroblastoma MN-1 altered, and CNS tumor with *BCOR-ITD*. DIG/DIA and infant-type hemispheric glioma may have similar pathological and radiological features like large size, cystic architecture, superficial /meningeal location, and possible ganglionic morphology. DIA/DIG mostly show grade 1 areas with foci of desmoplasia and molecular analysis showing *BRAF* alterations with MAPK pathway activation [38,45]. Supratentorial ependymoma may be confused with infant-type hemispheric glioma with focal ependymal differentiation. However, supratentorial ependymoma is characterized by clear cell morphology, negativity for oligodendrocyte transcription factor 2 (*OLIG2*), dot-like epithelial membrane antigen (*EMA*) positivity, and positivity for L1 cell adhesion molecule (*L1CAM*) and *p65/RELA* markers [46]. Astroblastoma is characterized by pseudorosettes and vascular hyalinization [47]. CNS tumors with *BCOR* ITD manifest branching arcuate capillary network, myxoid background, strong nuclear *BCOR* expression, and related molecular findings [48]. A concise overview of the differential diagnoses between pediatric diffuse glioma types and related conditions is presented in Table 1.

**Table 1:** Differential diagnosis

Pediatric diffuse glioma [A]	Differential diagnosis [B]	Similar features of type B	Differentiating features of type B
Diffuse astrocytoma, MYB/MYBL1 altered	Angiocentric glioma Adult-type diffuse glioma	Morphology and *MYB* gene involvement Overlapping morphology	Different fusion partner IDH mutation
Polymorphous low-grade neuroepithelial tumor of the young (PLNTY)	Oligodendroglioma (ODG)	Similar morphology	ODG is *CD34* negative, IDH & 1p/19q codeletion positive
Diffuse low-grade glioma, MAPK pathway altered	DENT Pilocytic astrocytoma	Overlapping morphology Overlapping morphology	Conspicuous histology IHC and molecular findings
Diffuse midline glioma (DMG) H3 K27-altered	Ganglioglioma	*H3K27* mutation	Presences of Rosenthal fibers and eosinophilic granular bodies
Diffuse hemispheric glioma H3 G34-mutant	Adult high-grade glioma	Overlapping morphology	Diffuse p53 positivity, Negative for *ATRX* and *OLIG2*
Diffuse pediatric-type high-grade glioma H3-wildtype and IDH-wildtype	Adult glioblastoma	High-grade morphology	*BRAF* mutation, *CDKN2A/CDKN2B* deletion and +7/-10 chromosomal abnormalities
Infant-type hemispheric glioma	Desmoplastic infantile ganglioglioma/astrocytoma (DIG/DIA)	Pathological & radiological features	Grade 1 areas, foci of desmoplasia, *BRAF* alterations

### Prognostic and therapeutic factors

Diffuse astrocytoma, MYB/MYBL1 altered, and angiocentric glioma are classified as WHO grade 1 tumors. These tumors have an indolent behavior, often resulting in patients becoming seizure-free after resection, with rare postoperative recurrence. Similarly, PLNTY are also considered grade 1 tumors and typically respond well to surgical excision. MAPK pathway-altered diffuse low-grade glioma rarely undergoes an anaplastic transformation. Novel innovations in targeted therapies like *BRAF* and *MEK* inhibitors may further alter the prognostic and predictive value of genetic alterations [3-6].

The prognosis of patients with PDHGG is usually poor, with a median survival of about one year. For *H3*-wildtype tumors, the median survival extends to approximately six years, with longer survival rates reported in adults and patients older than ten years [22]. Diffuse midline glioma (DMG) also has a poor prognosis, with a 2-year survival rate of less than 10% and a median survival of 10-12 months for the EGFR-mutant subtype. DMG with EZHIP amplification has a slightly longer survival. Poor prognosis is related to its location, which involves vital neural structures, making the surgical resection difficult. A clinical trial with GD2 (disialoganglioside) CAR-T (chimeric antigen receptor T) cell therapy is in process and seems effective [49]. The median survival of *DHG, G34* mutant is 22 months. *EGFR, CDK2*, and *MDM2* amplification are associated with worse prognosis. Identifying PDGFR mutation may open doors for new therapeutic regimens [18,50]. PDHGG tumors that are both *H3* and *IDH* wild type are aggressive, with the worst outcomes seen in tumors with *MYCN* amplification, intermediate outcomes with *PDGFRA* alterations, and better prognoses with *EGFR* alterations. *PDGFRA* and *EGFR* are considered potential therapeutic targets for the future [24]. WHO grading has not been assigned to infant-type hemispheric glioma. *ALK* fusion-positive tumors have a better prognosis than *ROS1* and *NTRK* fusion-positive tumors [24]. Using *RTK*-targeted therapeutic agents, such as Larotrectinib for *NTRK* fusion and Alectinib for *ALK* fusion, may alter the prognosis of tumors [51].

## CONCLUSION

PDLGG, a new category in the WHO CNS tumors classification 2021, comprises four tumors showing *MYB/MYBL1* fusion with a partner gene or MAPK pathway activating mutations involving primarily *BRAF* or *FGFR1*. However, this classification is still in its early stages and presents some confusion. Angiocentric glioma and diffuse astrocytoma MYB/MYBL1 altered tumors show overlapping histopathological features and both express *MYB* gene involvement. Similarly, PLNTY and diffuse glioma MAPK pathway-altered both present with *BRAF* or *FGFR* gene involvement with only limited microscopic differences. So, classification needs to be modified in the upcoming edition by integrating subtypes conforming to molecular pathology, which will be helpful for diagnostic and further therapeutic purposes.

PDHGG includes four new categories: two with mutations in the *H3* histone gene and two with *RTK* gene alterations. Each category has a differential diagnosis with other pediatric and adult gliomas, including glioblastoma and ganglionic tumors. Future updates may add new entities or merge existing ones based on the discovery of new molecular alterations in PDHGG.

The classification of CNS tumors is an evolving process, and future updates are expected to rely predominantly on molecular profiles. These updates will likely help identify novel therapeutic and prognostic markers, further advancing the understanding and treatment of these tumors.
